# Intramolecular Domain Movements of Free and Bound pMHC and TCR Proteins: A Molecular Dynamics Simulation Study

**DOI:** 10.3390/cells8070720

**Published:** 2019-07-13

**Authors:** Rudolf Karch, Claudia Stocsits, Nevena Ilieva, Wolfgang Schreiner

**Affiliations:** 1 Section of Biosimulation and Bioinformatics, Center for Medical Statistics, Informatics and Intelligent Systems (CeMSIIS), Medical University of Vienna, Spitalgasse 23, A-1090 Vienna, Austria; 2 Institute of Information and Communication Technologies (IICT), Bulgarian Academy of Sciences, Acad. G. Bonchev Str., Block 25A, 1113 Sofia, Bulgaria; 3 CERN-TH, Esplanade des Particules 1, 1211 Geneva, Switzerland

**Keywords:** molecular dynamics simulation, major histocompatibility complex, antigen, T-cell receptor, domain movements

## Abstract

The interaction of antigenic peptides (p) and major histocompatibility complexes (pMHC) with T-cell receptors (TCR) is one of the most important steps during the immune response. Here we present a molecular dynamics simulation study of bound and unbound TCR and pMHC proteins of the LC13-HLA-B*44:05-pEEYLQAFTY complex to monitor differences in relative orientations and movements of domains between bound and unbound states of TCR-pMHC. We generated local coordinate systems for MHC α1- and MHC α2-helices and the variable T-cell receptor regions TCR V_α_ and TCR V_β_ and monitored changes in the distances and mutual orientations of these domains. In comparison to unbound states, we found decreased inter-domain movements in the simulations of bound states. Moreover, increased conformational flexibility was observed for the MHC α2-helix, the peptide, and for the complementary determining regions of the TCR in TCR-unbound states as compared to TCR-bound states.

## 1. Introduction

The interaction between T-cell receptors (TCR) and antigenic peptides (p) presenting major histocompatibility complexes (pMHC) is a crucial step in adaptive immune response [1]. It triggers the generation of cell-mediated immunity to pathogens and other antigens. The response is driven by TCRs specifically recognizing antigenic peptides bound to and presented by MHC (pMHC) of infected or transformed cells. MHC class I molecules are membrane bound proteins on the surface of antigen presenting cells consisting of an α-chain and a β2-microglobulin. Short fragments of antigens are presented in a pocket formed by the α1 and α2 domains. The MHC-bound peptides are presented to the TCR for examination. The TCR itself is a heterodimer on the surface of T-cells and is formed by an α-chain and a β-chain (each chain containing a variable and a constant region) [2]. The binding site of the T-cell receptor is constituted by the two variable regions TCR V_α_ and TCR V_β_, which specifically recognize the α-helices of the MHC peptide-binding groove as well as parts of the antigen [3]. The interaction of TCR and pMHC is weak but highly specific, therefore being able to distinguish self-antigens from foreign peptides [1]. Additional molecules and co-receptors, e.g., the CD8, a T-cell surface glycoprotein, help establish a stable, specific and sensitive interaction. Correct binding of the TCR to pMHC induces a kinase mediated signaling cascade and activation of the T-cell immune response.

The TCR approaches the MHC in a diagonal orientation relative to the pMHC-binding groove, driven by long-range electrostatic interactions and low affinity binding events [4,5]. During the binding process the complementarity determining regions (CDRs) of the TCR V_α_ (CDR1α, CDR2α, CDR3α) and TCR V_β_ domains (CDR1β, CDR2β CDR3β) are positioned over the N- and C-terminus of the peptide and form contacts [6,7]. Local conformational readjustment occurs after binding of the TCR to the pMHC [8,9,10]. Complexation of TCR- and pMHC-molecules takes place via hydrogen bonds, salt links, and van der Waals interactions with different relative contributions [4].

Protein-protein interfaces within crystal structures of TCR-pMHC complexes have been evaluated in numerous studies and provided insight into the molecular mechanism behind the interaction and recognition mechanism of TCR with the pMHC [6,11,12] but lack information about the intermolecular approach and reorientation. Details of the binding and spatial rearrangements of the TCR-pMHC interaction on an atomistic scale remain to be uncovered.

Molecular dynamics (MD) is a powerful tool to investigate dynamic molecular processes and movements by solving Newton’s equations of motion and has long been an adequate device to complement experimental crystallographic and biophysical studies on proteins. The first MD studies focused on pMHC complexes and were of comparably short duration due to the limitations in computational resources. Early MD simulations on a very short timescale ≤ 0.6 ns by Toh et al. [13] suggested that antagonist peptides bound to HLA-DR4, an MHC class II, display greater flexibility in the putative TCR binding regions compared to agonist peptides. Similar results on epitope flexibility, albeit with MD simulations of 100 ns duration, were reported by Reboul et al. [14]. Zacharias and Springer [12] studied the conformational flexibility of the MHC Class I peptide-binding region (α1- α2 domain) in peptide bound and free states with several independent MD simulations of 26 ns each and reported an increased conformational heterogeneity in the absence of a peptide ligand. As to TCR-pMHC interactions, Wolfson et al. [15] performed a series of 15 ns MD simulations studying peptide and TCR mutations and found that the footprint of the TCR on the pMHC is insensitive to mutations of the TCR peptide-binding loops, but peptide mutations can make multiple local changes to TCR-pMHC interface dynamics and interactions. A similar trend was observed in 20 ns long MD simulations of the LC13 T-cell receptor in complex with HLA-B*0801 and the Epstein-Barr virus (EBV) derived peptide FLRGRAYGL [16]. The results of this study revealed a high mobility of the CDR-loops of the TCR as well as the presence of two conformational clusters in the peptide’s structure, underlying the backbone flexibility of the peptide. Very recently, Tedeschi et al. [17] used MD simulations to complement their experimental studies on an unusual placement of an EBV epitope in the binding groove of an ankylosing spondylitis (AS)-associated HLA-B27 allele that allowed CD8+ T cell activation and inferred from computational analysis that the strongest risk factor for AS, i.e., B*2705, is able to elicit anti-viral T cell immune-responses even when the binding groove might be partially occupied by the epitope. In the present study, we performed MD simulations of bound and unbound TCR-pMHC configurations of a LC13-HLA-B*44:05-pEEYLQAFTY complex in order to evaluate if and how intramolecular domain movements and relative orientations (namely between TCR V_α_ and TCR V_β_, and MHC α1 and MHC α2) change due to complexation of TCR with pMHC molecules.

## 2. Methods and Materials

### 2.1. System and Start Configurations

**Figure 1:**
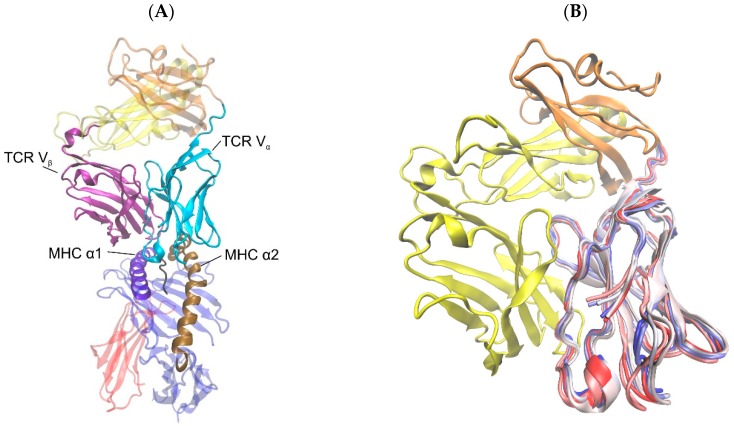
Cartoon representations of TCR and pMHC molecules. (**A**) TCR-pMHC complex from the crystal structure 3KPS with highlighted domains TCR V_α_ (cyan), TCR V_β_ (purple), MHC α1 (violet), and MHC α2 (ochre), TCR constant domain α (orange), TCR constant domain β (yellow), MHC α3 (blue), MHC β-microglobulin (red), peptide (black). (**B**) TCR alone with overlaid snapshots of domain TCR V_α_ at every 8 ns from a 40 ns simulation run of the TCR-pMHC complex to illustrate the extent of domain movements. The beginning of the trajectory appears in red, the middle in white, and the end in blue. The figures were produced using VMD [22].

We used a set of three different start configurations: In the first set MD simulations were started from the crystal structure of a complex consisting of a LC13 TCR, a self-peptide (EEYLQAFTY) from the ATP binding cassette protein ABCD3, and a MHC of the HLA-B*44:05 type (PDB-ID: 3KPS, see Figure 1). This system was chosen because it is available at a high resolution of 2.7 Å. Moreover, Macdonald et al. [18] determined binding characteristics and immunogenicity of three MHC alleles (HLA-B*44:02, HLA-B*44:03, and HLA-B*44:05) in complex with the ABCD3 nine-mer peptide and the LC13 TCR and Ferber et al. [19] analyzed TCR binding orientations over pMHC. In previous papers we used the 3KPS system for geometric analyses of alloreactive HLA α-helices [11,20] as well as for finding semi-rigid domains in MD simulations [21]. In the present study we consider differences in the dynamics of intramolecular domains between bound and unbound states of the 3KPS system. In the second and third set of configurations, simulations were started from isolated, i.e., unbound TCR- and pMHC-molecules as derived from the original 3KPS crystal structure.

### 2.2. Molecular Dynamics Simulations

MD simulations were run in an explicit water solvent using GROMACS 5.1.1 [23]. We used the amber99sb-ildn force field [24] to simulate the protein placed in a rhombic dodecahedral box with 2 nm minimum distance between the protein atoms and the box boundary. The whole system contains approximately 397,000 atoms, consisting of 826 protein residues and approximately 128,000 solvent molecules. Based on the SPC water model [25] the protein was solvated and neutralized by replacing solvent molecules with Na^+^ and Cl^−^ ions to reach a salt concentration of 0.15 mol/L.

After solvation the whole complex was energetically minimized using the steepest-descent approach with 10,000 steps. For equilibration we set the temperature of the system to 310 K and the equations of motion were followed for 100 ps using position restraint MD and a Berendsen-thermostat with a time constant of 0.1 ps. This equilibration under NVT conditions (constant number of particles, volume and temperature) was followed by a 100 ps equilibration in an NPT ensemble (constant number of particles, pressure and temperature) to control the pressure by a Berendsen-barostat set to 1 bar with a time constant of 1.0 ps.

The MD production runs were carried out with a time-step of 4 fs using the LINCS constraint algorithm for all bonds and virtual sites for hydrogen atoms. For the Van der Walls and Coulomb interactions a cut-off radius of 1.4 nm was applied. Long-range Coulomb interactions were computed using the particle-mesh Ewald method with a maximum grid spacing of 0.12 nm for the FFT and an interpolation order of 4. Velocity-rescaling temperature coupling was set to 310 K and Berendsen isotropic pressure coupling was set to 1 bar. Coordinates were written to the trajectory file on hard disk every 40 ps, resulting in 1000 frames for each run. This procedure was repeated for each run in the set of initial configurations. We performed 30 independent MD simulation runs with different initial velocities and with a length of 40 ns each: 10 runs for the TCR-pMHC complex (bound configurations), 10 runs for a free pMHC molecule, and 10 runs for a free TCR molecule (unbound configurations). For reasons of comparison, two additional runs of 400 ns in length (one for the free pMHC- and the other for the free TCR-molecule) and one 144 ns run for the TCR-pMHC complex were performed. Prior to the analysis of the trajectories, translational and rotational motions of the protein relative to the equilibrated structure were removed.

### 2.3. Distance between Domains

One commonly used method among many others [26,27,28] to characterize relative movements of intramolecular domains is the calculation of distances between groups of atoms (domains) as a function of simulation time. We evaluated the distances *d* between the geometric centers of domains, based on the C_α_ atoms in the protein backbone.

### 2.4. Relative Orientation of Two Intramolecular Domains

Based on the methods described previously [29] we monitored the relative orientations of intramolecular domains during the time-course of the simulations. For the definition of domains we used the C_α_ atoms of the protein backbone. As a first pair of domains we chose the two epitope binding domains TCR V_α_ (104 residues) and TCR V_β_ (117 residues), both involved in the formation of the interface with pMHC. As a second pair we considered the antigen presenting sites of MHC, namely MHC α1 (25 residues) and MHC α2 (31 residues).

**Figure 2:**
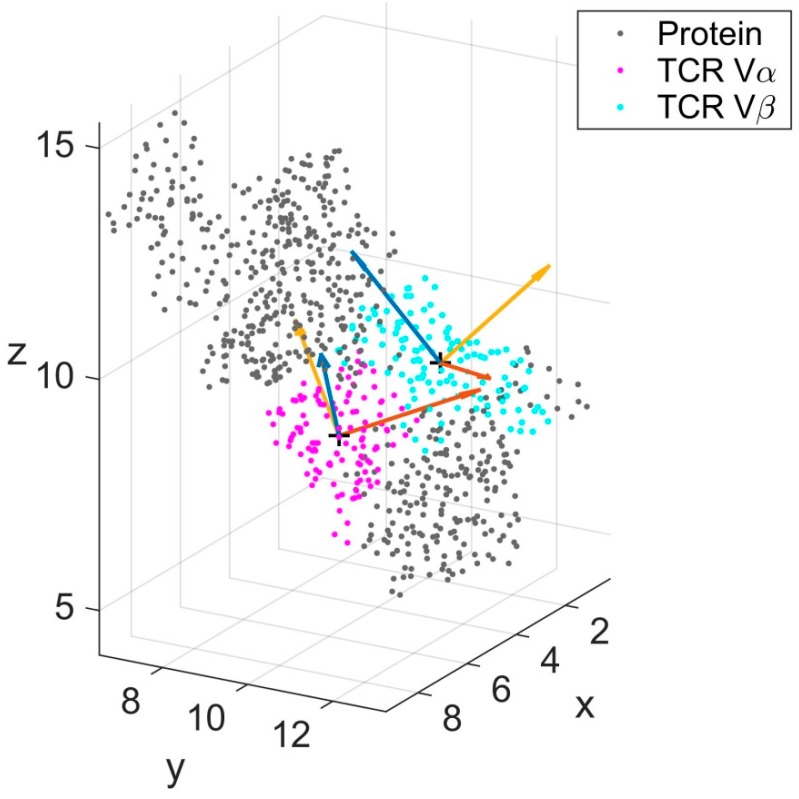
Schematic representation of TCR V_α_ (magenta) and TCR V_β_ (cyan) with local eigenvectors. The remaining parts of the complex (labelled Protein) are shown in gray. Eigenvectors (**v**_1_, **v**_2_, **v**_3_ and **w**_1_, **w**_2_, **w**_3_) are displayed for a single frame of the trajectory and colored blue (**v**_1_, **w**_1_), orange (**v**_2_, **w**_2_) and yellow (**v**_3_, **w**_3_). Units of the coordinate axes are in nm.

During an MD simulation the atoms within each domain move according to the equations of motion and thus their relative orientation changes from time step to time step. Principle component analysis (PCA) is commonly used to analyze the motions of domains within the conformations obtained during MD simulations [26]. PCA as such yields eigenvectors unique in directions but ambiguous in orientation. To arrive at standardized eigenvectors allowing for comparison between different MD-runs we chose the crystal structure as a reference frame and re-oriented (if necessary) the resulting eigenvectors after performing the PCA [29]. The three orthonormal eigenvectors from the PCA were used to define an eigenvector matrix to serve as a local coordinate system, moving together with the domain, see Figure 2. For each frame of an MD simulation we monitored the relative orientation between local coordinate systems of the domains.

To actually characterize the relative orientations of domains and their changes over time, we computed the cosines between the corresponding eigenvectors pairs (**v**_1_, **w**_1_), (**v**_2_, **w**_2_) and (**v**_3_, **w**_3_) of the respective local coordinate systems.

### 2.5. Domain Deformations and Fluctuations

To quantify intra-domain deformations we computed the RMSD (root-mean-square deviation) of each frame with respect to the first frame of each trajectory, considering only the C_α_ atoms within the respective domain. The root mean square deviation RMSD(*t*) of a group of atoms in a molecule at time *t* with respect to a given reference structure is a measure of deformations (deviations in shape), i.e., the square root of averaged squared distances between the atom positions at time *t* and the positions in the reference structure. Here, the starting structure of the production runs (i.e., the first frame of the trajectory) was chosen as the reference structure. As such, RMSD(*t*) is a statistical measure of the distance of a group of atoms at a particular time *t* with respect to the same group in the reference structure and was calculated as:

(1) $$\mathrm{RMSD}\left(t\right)=\sqrt{\frac{1}{N}\overset{N}{\underset{i=1}{\sum }}{\left|r_{i}\left(t\right)-r_{i}^{\mathrm{ref}}\right|}^{2}}$$

where **r***_i_*(*t*) is the position of atom *i* at time *t* after least square fitting to the reference structure, *N* is the total number of atoms in the group of atoms considered, and **r***_i_*^ref^ is the position of atom *i* in the reference structure. On the other hand, the root mean square fluctuation RMSF(*i*) is a statistical measure of the deviation between the position of atom *i* (or a group of atoms, e.g., a residue) and some reference position **r***_i_*^ref^:

(2) $$\mathrm{RMSF}\left(i\right)=\sqrt{\frac{1}{T}\overset{T}{\underset{t_{j}=1}{\sum }}{\left|r_{i}\left(t_{j}\right)-r_{i}^{\mathrm{ref}}\right|}^{2}}$$

where *T* is the time interval over which the average is taken and *t_j_* is the time of frame *j* during the simulation of the respective trajectory. Here, we have chosen the time-averaged position of atom *i* as the reference position, i.e., **r***_i_*^ref^ = <**r***_i_*>. Whereas the RMSD(*t*) is an average taken over all atoms within a group for a specific time *t*, the RMSF(*i*) is an average over time for a specific atom *i*.

## 3. Results

### 3.1. Relative Movements between TCR V_α_ and TCR V_β_

**Figure 3:**
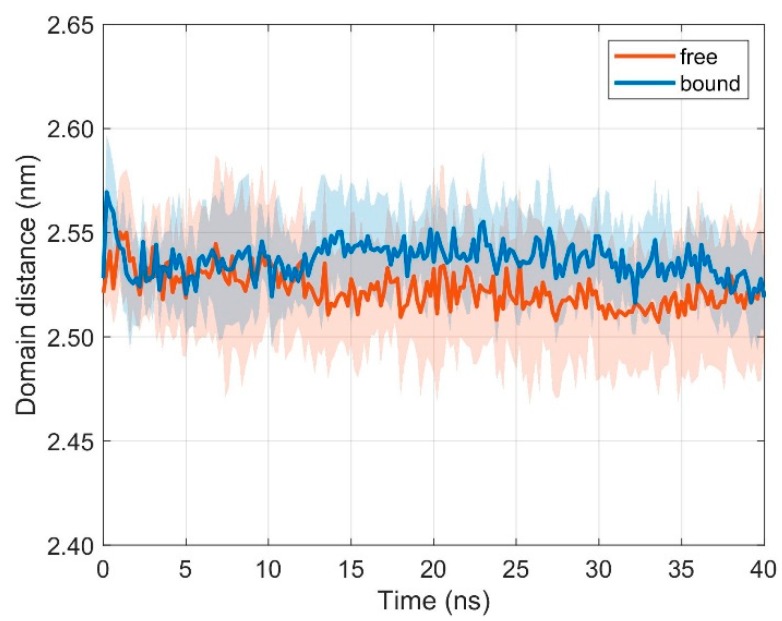
Time course of inter-domain distance *d* between domains TCR Vα and TCR Vβ. Mean ± SD for unbound (orange) and bound (blue) configurations from ten 40 ns runs with distance evaluation every 0.2 ns.

The variable regions TCR V_α_ and TCR V_β_ interact with the pMHC surface and are therefore important during TCR-pMHC recognition. Interestingly, if TCR and pMHC are free (i.e., unbound), the distance *d* between the domains TCR V_α_ and TCR V_β_ is not distinctively different from the bound configurations (results from ten 40 ns runs): < *d***_free_** > = 2.523 nm, < *d***_bound_** > = 2.537 nm, ∆< *d* > = 0.014 nm (difference of mean distances < *d* > between bound and unbound configurations), σ_free_ = 0.005 nm (standard deviation of distances for unbound configurations), and σ_bound_ = 0.002 nm (standard deviation of distances for bound configurations), see Figure 3.

**Figure 4:**
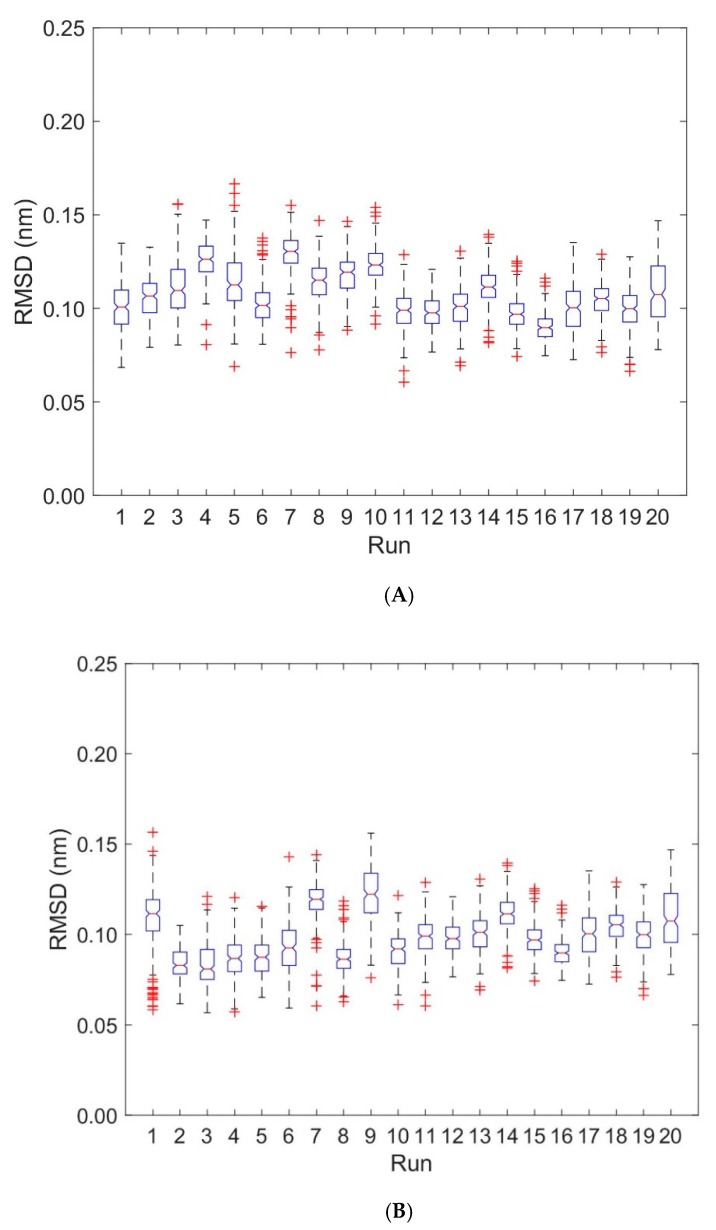
Boxplots of RMSD for TCR V_α_ and TCR V_β_. RMSD-values are computed for every 0.2 ns with respect to the first frame of each trajectory. Run numbers 1 to 10 correspond to unbound states, 11 to 20 to bound states. (**A**) RMSD for TCR V_α_ domain. (**B**) RMSD for TCR V_β_ domain.

Deformations within each domain can be characterized by RMSD-values. Figure 4 shows boxplots of RMSD-values computed with respect to the first frame of each trajectory for the unbound (runs 1 to 10) and bound states (runs 11 to 20). Deformations within TCR V_α_ and TCR V_β_ are virtually unaffected by the binding of the pMHC und TCR molecules (see Figure 4, Table 1 and Table 2): For TCR V_α_ the overall mean ± SD of RMSD-values from ten 40 ns runs were 0.115 ± 0.002 nm for the unbound states and 0.101 ± 0.002 nm for the bound states. For TCR V_β_ the respective values were 0.096 ± 0.003 nm (unbound) and 0.101 ± 0.002 nm (bound).

**Figure 5:**
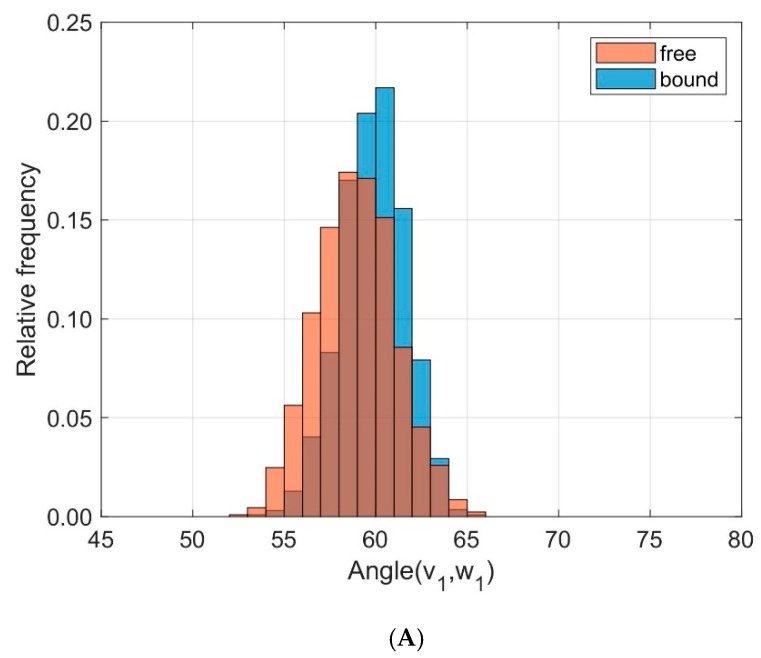

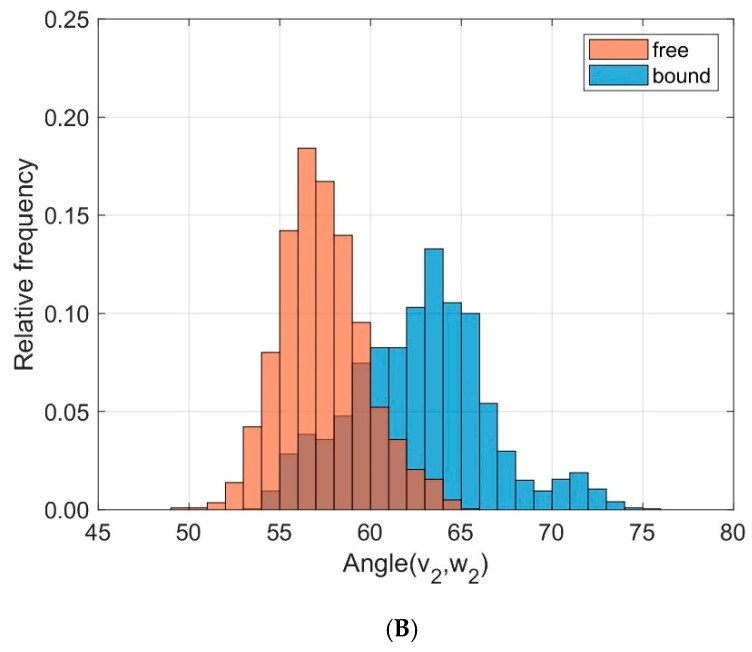
Frequency distribution of angles between corresponding principal component eigenvectors of domains TCR V_α_ and TCR V_β_ for bound (blue) and unbound states (orange). (**A**): Angles between eigenvectors **v**_1_, **w**_1_ corresponding to the main extensions (*D* = 0.215); (**B**): Angles between **v**_2_, **w**_2_ (*D* = 0.643).

We characterized the relative orientation of the domains TCR V_α_ and TCR V_β_ by the directional cosines between eigenvectors attached to each domain and sampled over time for all trajectories as outlined in the methods section and in our previous paper [29], see Figure 5. The eigenvectors **v**_1_ and **w**_1_ correspond to the main extensions of the respective domain. To quantify the differences between bound and unbound states in the frequency distributions of the respective angles α between **v**_1_ and **w**_1_ we used the test statistic *D* of the Kolmogorov Smirnov test. For **v**_1_ and **w**_1_ the results from ten 40 ns runs were *D* = 0.215, < α(**v**_1_,**w**_1_) >_free_ = 58.96°, σ_free_ = 2.15°, < α(**v**_1_,**w**_1_) >_bound_ = 59.88°, σ_bound_ = 1.75°. Larger differences were observed for the angles β between **v**_2_ and **w**_2_: *D* = 0.643, < β(**v**_2_,**w**_2_) >_free_ = 57.38°, σ_free_ = 2.36°, < β(**v**_2_,**w**_2_) >_bound_ = 62.78°, σ_bound_ = 3.78°.

These results may be summarized as follows: The domains TCR V_α_ and TCR V_β_ remain relatively unchanged in their main orientation after complexation of TCR and pMHC, as seen by the small changes of the angles between **v**_1_ and **w**_1_ (58.96° on average for the unbound state versus 59.88° in the bound state), see Figure 5A. An additional change in orientation is due to a rotation around these major axes as reflected in the variation of the angles β between **v**_2_ and **w**_2_. As a result of complexation their mean values change from 57.38° for the unbound states to 62.78° for the bound states, see Figure 5B.

### 3.2. Relative Movements between MHC α1 and MHC α2

**Figure 6:**
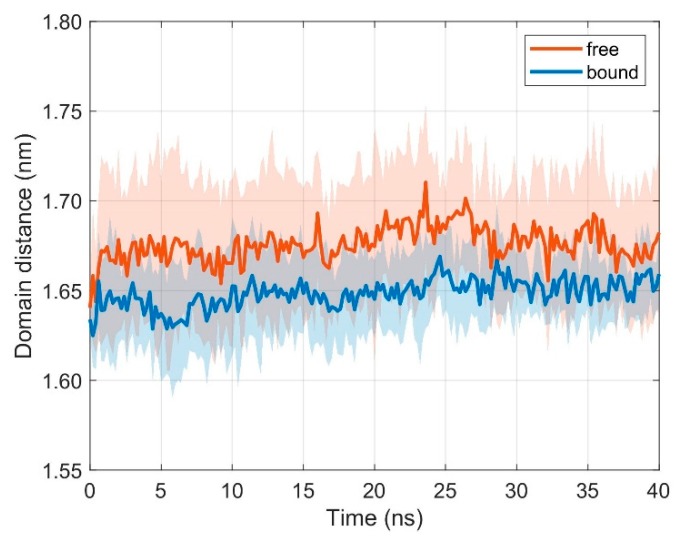
Time course of inter-domain distance *d* between domains MHC α1 and MHC α2. Mean ± SD for unbound (orange) and bound (blue) configurations from ten 40 ns runs with distance evaluation every 0.2 ns.

The two α-helices of the MHC, together with a β-floor, form the peptide binding cleft that presents antigen fragments to be examined by the TCR. Together with the variable domains of the TCR, TCR V_α_ and TCR V_β_, they are most important for the formation of the TCR-pMHC complex and for a successful initiation of the immune response. We thus also monitored intramolecular movements of the two α-helices in the free (i.e., unbound) and bound states. If TCR and pMHC are unbound, the distance *d* between domains MHC α1 and MHC α2 practically does not change with respect to the bound state (results from ten 40 ns runs): < *d***_free_** > = 1.676 nm, < *d***_bound_** > = 1.649 nm, ∆< *d* > = 0.028 nm (difference of mean distances < *d* > between unbound and bound configurations), σ_free_ = 0.004 nm (standard deviation of distances for unbound configurations), and σ_bound_ = 0.004 nm (standard deviation of distances for bound configurations), see Figure 6.

**Figure 7:**
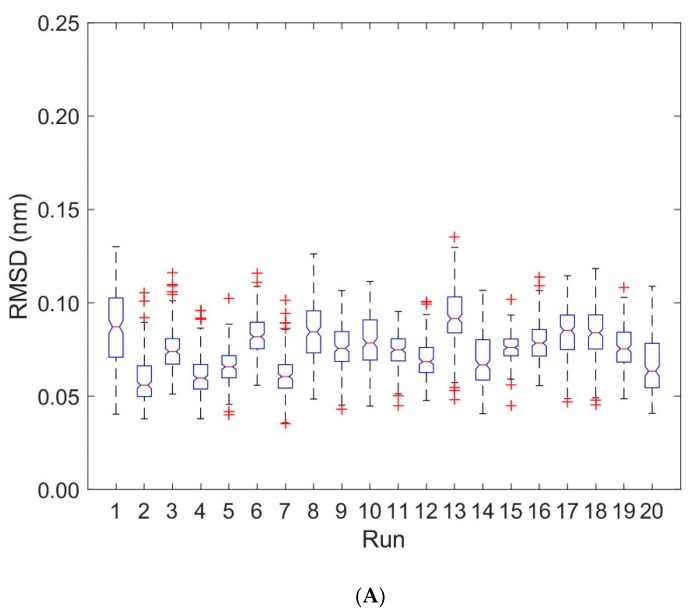

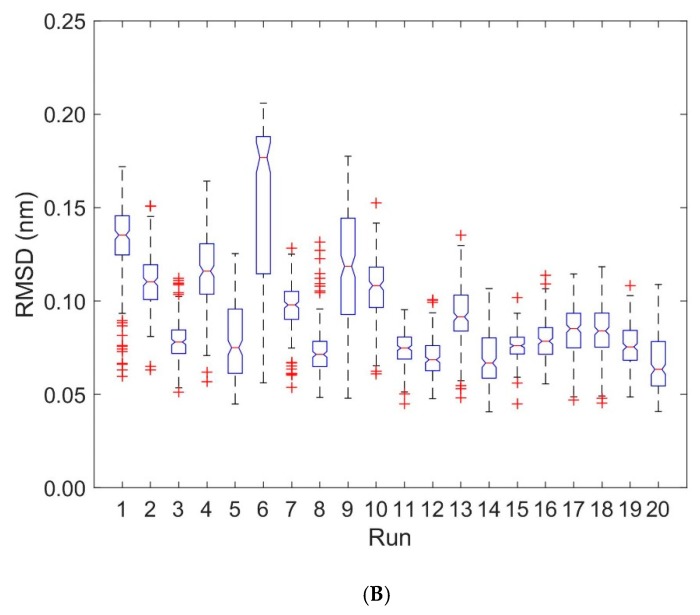
Boxplots of RMSD for MHC α1 and MHC α2. RMSD values are computed every 0.2 ns with respect to the first frame of each trajectory. Run numbers 1 to 10 correspond to unbound states, 11 to 20 to bound states. (**A**): RMSD for the MHC α1 domain. (**B**): RMSD for the MHC α2 domain.

We computed RMSD-values of each domain MHC α1 and MHC α2 with respect to the first frame of each trajectory for the unbound (runs 1 to 10) and bound states (runs 11 to 20), see Figure 7, Table 1 and Table 2: For the MHC α1 domain, the boxplots of RMSD do not show any systematic dependence on the binding state of pMHC and TCR (the overall mean ± SD of RMSD-values from ten 40 ns runs were 0.073 ± 0.003 nm for the unbound states and 0.077 ± 0.003 nm for the bound states). In contrast to the MHC α1 domain, the RMSD-values of the MHC α2 domain exhibited a pronounced difference between unbound and bound configurations: The unbound state not only shows larger RMSD-values than the bound state (mean 0.107 nm versus 0.077 nm), but also a three-fold variability in RMSD-values (SD 0.009 nm versus 0.003 nm).

**Figure 8:**
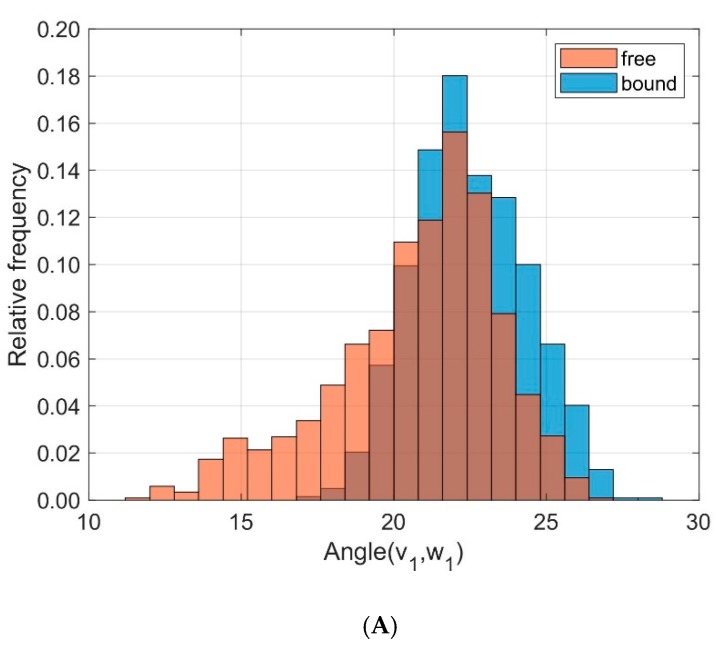

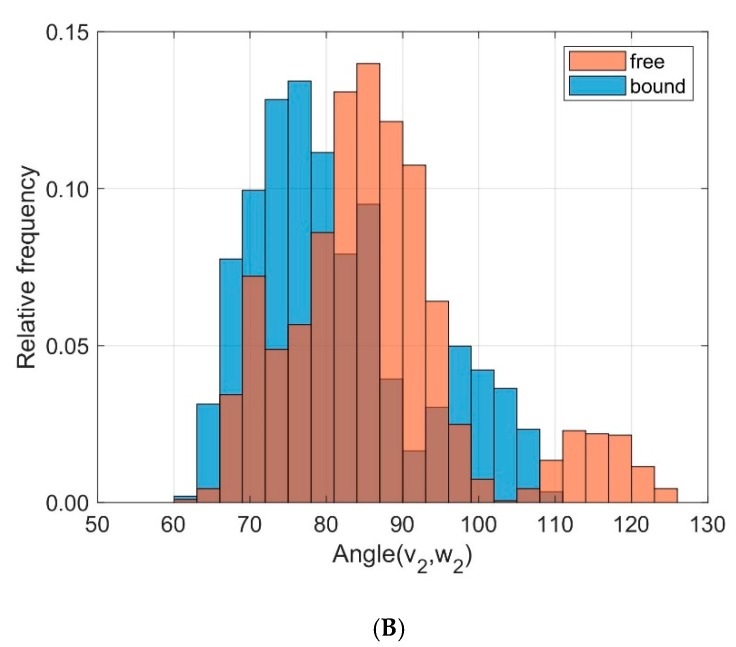
Frequency distribution of angles between corresponding principal component eigenvectors of domains MHC α1 and MHC α2 for bound (blue) and unbound states (orange). (**A**): Angles between eigenvectors **v**_1_, **w**_1_ corresponding to the main extensions (*D* = 0.255); (**B**): Angles between **v**_2_, **w**_2_ (*D* = 0.290).

To monitor the relative orientation between the helical domains MHC α1 and MHC α2 we calculated directional cosines between corresponding eigenvectors and examined their distributions over time, see Figure 8. The angles α between the eigenvectors **v**_1_ and **w**_1_ (corresponding to the main extensions of the domains) were slightly different between unbound and bound states (results from ten 40 ns runs): *D* = 0.255, < α(**v**_1_,**w**_1_) >_free_ = 20.75°, σ_free_ = 2.74°, < α(**v**_1_,**w**_1_) >_bound_ = 22.50°, σ_bound_ = 1.85°. More pronounced differences and larger fluctuations were observed for the angles β between **v**_2_ and **w**_2_: *D* = 0.290, < β(**v**_2_,**w**_2_) >_free_ = 86.57°, σ_free_ = 12.26°, < β(**v**_2_,**w**_2_) >_bound_ = 81.16°, σ_bound_ = 11.06°. These eigenvectors **v**_2_ and **w**_2_ point away from the axis of the helices at right angles and indicate a larger variation in the relative orientation between MHC α1 and MHC α2 in the unbound state as compared to the bound state.

## 4. Discussion

Crystallographic studies of TCR-pMHC complexes have delivered important insights regarding the structural components involved in stabilizing the TCR-pMHC complex. Despite the huge success and the valuable contributions of these studies, they provide only a limited static view. On the other hand, molecular dynamics simulations allow for following the dynamics of single atoms or groups of atoms with a time-resolution in the order of pico-seconds. Although the interaction between a TCR and a pMHC is weak (10^3^–10^5^ M^−1^ s^−1^ [30]), it represents an important initial step in T-cell activation. Detailed studies on protein-protein complexes suggest that the binding process of protein molecules can be divided into three phases [31]: A first and short phase of diffusion (initial contacts form within 2 ns) is followed by an intermediate phase where most native contacts are established (between 50–200 ns). In the last phase the final stereospecific complex is formed (this takes hundreds of nanoseconds). Various studies have reported conformational changes at the TCR-pMHC binding interface in the course of complex formation, in particular at the CDRs and peptides and less at the MHC [32,33,34]. In the current work, we present molecular dynamics simulations to study the differences in intramolecular domain movements between bound and unbound states of a specific TCR-pMHC complex.

For the analyses of domain orientations, we attached local coordinate systems (PCA-eigenvectors) to the domains and calculated the cosines between corresponding eigenvectors. Based on three sets of independent simulations with different initial configurations (TCR-pMHC bound, TCR unbound, pMHC unbound) we could show that binding of TCR to pMHC decreases the flexibility in the inter-domain movements, in particular the dynamics of TCR V_α_ relative to TCR V_β_ and of MHC α1 relative to MHC α2, see the distribution of angles between corresponding principal component eigenvectors in Figure 5 and Figure 8**.**

**Figure 9:**
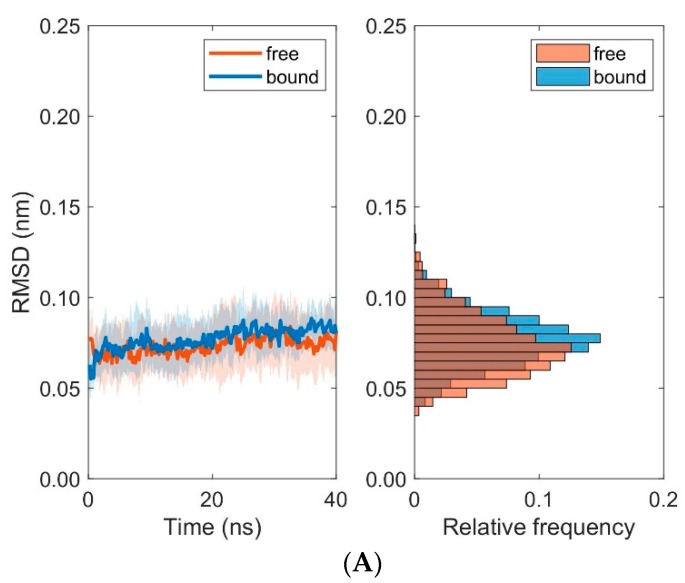

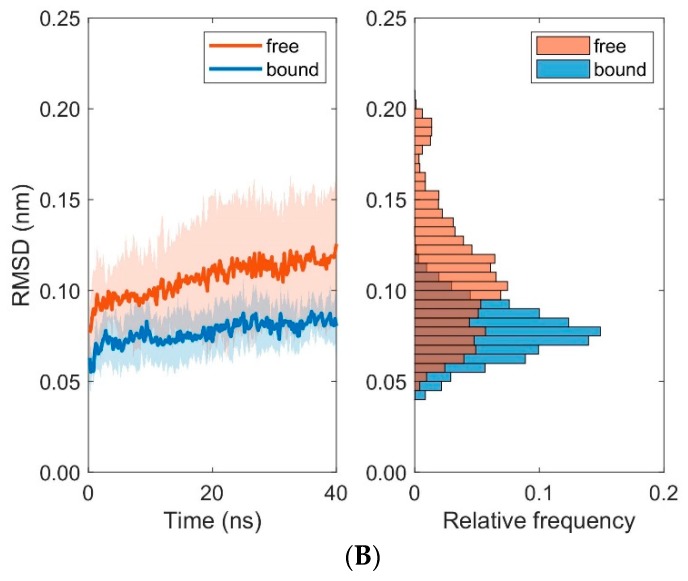
Time course and distribution of RMSD-values for domains MHC α1 (**A**) and MHC α2 (**B**). Time-series (left panels, mean ± SD) and histograms (right panels) display pooled RMSD-values from ten 40 ns MD simulations each for bound and unbound (free) states.

**Figure 10:**
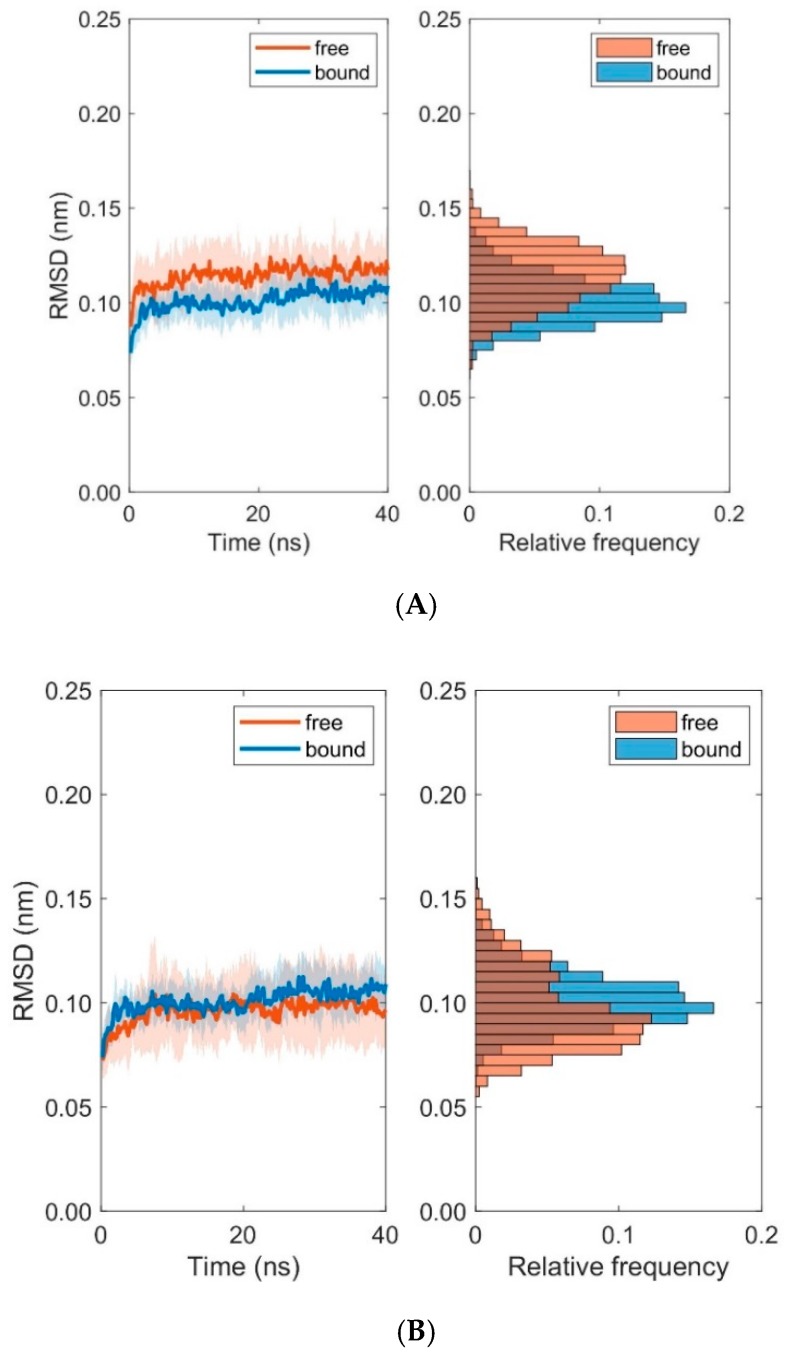
Time course and distribution of RMSD-values for domains TCR V_α_ (**A**) and TCR V_β_ (**B**). Time-series (left panels, mean ± SD) and histograms (right panels) display pooled RMSD-values from ten 40 ns MD simulations each for bound and unbound (free) states.

**Figure 11:**
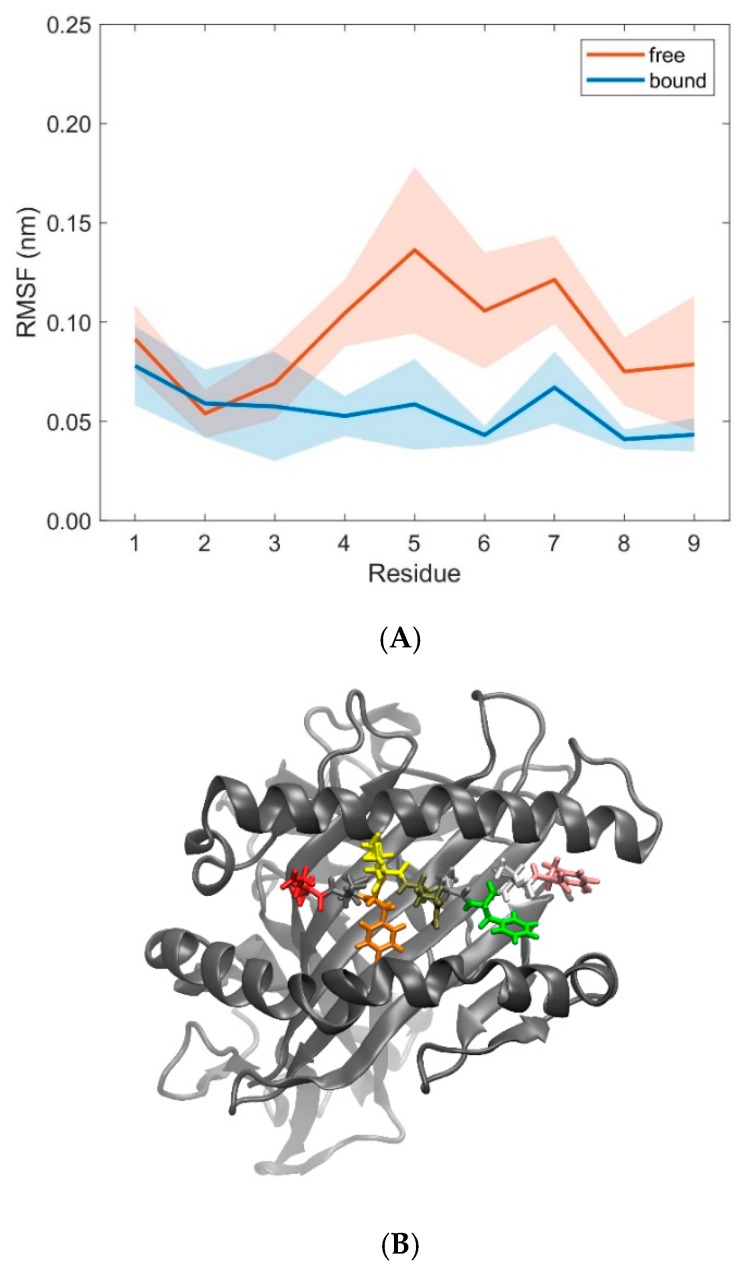
(**A**) RMSF-values (mean ± SD) of MHC peptide residues in free (orange) and bound (blue) states calculated from 10 pooled 40 ns MD simulations each for unbound (free) and bound states. (**B**) Cartoon representation of the 3KPS MHC binding groove showing the lateral α1- and α2-helices and the β-sheet (grey) together with the peptide (displayed as licorice) consisting of nine residues (position 1: Glu, red; position 9: Tyr, pink) as obtained from a snapshot of a 40 ns MD simulation of a TCR-free pMHC. The figure in panel B was produced using VMD [22].

Further examination of domain RMSD-values revealed that MHC α2-dynamics clearly depends on the binding states (i.e., bound versus unbound) of TCR and pMHC molecules, see Figure 9. Conformational flexibility, as characterized by the RMSD-values of MHC α2, was distinctively larger in the unbound states as compared to the bound states. On the contrary, differences between bound and unbound states in RMSD-values of the TCR domains V_α_ and V_β_ were considerably less pronounced, see Figure 10. The decrease of domain RMSD-values of MHC α2 is consistent with the dynamics of the peptide placed in the binding groove between the two MHC α-helices: Root mean square fluctuations (RMSF) of individual residues of the peptide during the course of the simulations show distinctively larger values in the unbound as compared to the bound states (Figure 11). In particular, central amino acid residues at positions 4–7 within the peptide (Leu, Gln, Ala, Phe) display reduced dynamic flexibility upon binding of the pMHC with the TCR, in agreement with previous studies of various peptide/MHC-I complexes and TCRs [35,36], see [6,37,38,39] for reviews. On the other hand, the N- and C-termini of positions 1 and 9 in the peptide are much less influenced by TCR-binding due to the network of hydrogen bonds from the β-sheet and from the lateral α-helices which stabilize the peptide binding domain [12]. Macdonald et al. [18] emphasized the importance of the C-terminal region (p6–p8) of the peptide for TCR recognition: In particular, the small p6 (Ala) and p8 (Thr) residues enabled the aromatic p7 (Phe) residue to protrude within a central pocket of the TCR, sandwiched between Tyr31α and Tyr100β; the p6-Ala made interactions with the CDR3α and the CDR3β loop, while p8-Thr and p7-Phe formed contacts with the CDR3β loop.

**Figure 12:**
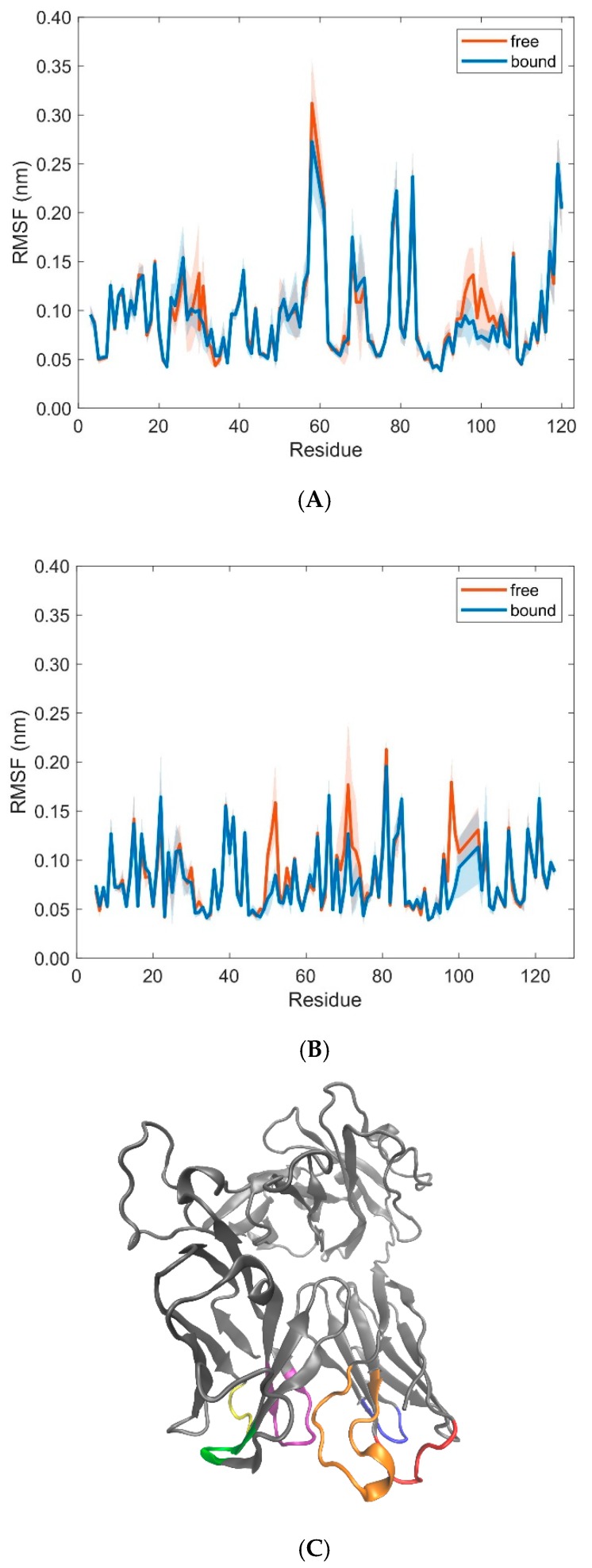
RMSF-values (mean ± SD) of TCR residues in the variable domains V_α_ (**A**) and V_β_ (**B**) in free (orange) and bound (blue) states calculated from 10 pooled 40 ns MD simulations each for unbound (free) and bound states. (**C**) Cartoon representation of the 3KPS TCR (grey) together with colored CDR loops CDR1α (24–31, red), CDR2α (48–55, blue), CDR3α (93–104, orange), CDR1β (26–31, yellow), CDR2β (48–55, green), CDR3β (95–107, purple) as obtained from the crystal structure of the 3KPS system. The figure in panel C was produced using VMD [22].

Residues in the variable domains V_α_ and V_β_ of the T-cell receptor show less pronounced differences in RMSF-values between free and bound states, with the exception of residues 30 (Thr), 31 (Tyr), 58 (Arg), 96–98 (Gly, Gly, Thr), 100 (Tyr), and 102 (Gly) in the TCR V_α_ domain and residues 50–52 (Gln, Asn, Glu), 71–73 (Glu, Gly, Ser), 98–100 (Gln, Ala, Tyr), and 105 (Glu) in the TCR V_β_ domain (Figure 12). Apart from residue 58 in the V_α_ domain and residues 71–73 in the V_β_ domain, all the other residues with a distinctively larger RMSF-value in the unbound states are part of the CDR-loops CDR1α (30, 31), CDR3α (96–98, 100, 102), CDR2β (50–52), and CDR3β (98–100, 105). These results are in agreement with several studies that have found a reduced mobility of the TCR [6,40] and conformational changes in the CDR-loops [4,7,41] upon formation of the TCR-pMHC complex. In particular, we observed a structural change between the free and bound states in the CDR3α loop of residues 95–97 (Ala, Gly, Gly) from a 3_10_-helix (free) to a coil (bound), where Gly96α and Gly97α form interactions with Arg62 and Ile66 of the MHC α1 helix, respectively [18] (see supplementary Figure S1). As to the type of interactions between CDR-loops and pMHC, Macdonald et al. [18] reported predominantly van der Waals interactions and H-bonds, including one salt bridge between Glu52β of CDRβ2 and Arg79 of the α1 helix. These crystallographic studies have also shown that CDR1α and CDR2α loops make contacts with the α2 helix of pMHC, whereas contacts to the α1 helix are established by the CDR2β and CDR3α loops (CDR3α also makes interactions with the α2 helix and the peptide). While the CDR1β loop participates only minimally in the TCR-pMHC interactions, the contacts with the peptide are dominated by the CDR3β loop, which sits centrally above the peptide-binding cleft, adjoining the CDR3α loop (see also Figure 12C).

Only minor changes were observed in the inter-domain distance between TCR V_α_ relative to TCR V_β_ and MHC α1 relative to MHC α2 as a consequence of binding. Fluctuations within domains (TCR V_α_, TCR V_β_, MHC α1, and MHC α2) are virtually unaffected by the binding state, see Figure 4 and Figure 7. In addition, angles between corresponding eigenvectors of MHC α1 and MHC α2 show larger variation in the unbound states, indicating a wider range of the groove between the two α-helices, see Figure 5 and Figure 8.

A study based on static crystallographic data [42] demonstrated that the angle between TCR V_α_ and TCR V_β_ domains varies depending on the presence of peptides, whereby domains show larger variations in the unbound state and smaller variations in the bound state of the TCR. These findings are consistent with our MD-based results regarding the angles between vectors **v**_1_ and **w**_1_.

As to the design of our MD experiments, we performed 10 independent 40 ns runs for each of the 3 configurations studied (pMHC free, TCR free, TCR-pMHC complexed). This relatively short simulation length was chosen because it is more efficient (in terms of sampling the conformational space) to run various short independent MD simulations than a single long one [43], see, e.g., the variations of RMSD-values between the runs in the boxplots of Figure 7. On the other hand, RMSD mean values as calculated from ten pooled 40 ns runs were consistent with the respective values obtained from one 144 ns run (TCR-pMHC complex) und two 400 ns runs (TCR and pMHC free), see run 11 in Table 1 and Table 2.

There are several limitations in the present study, in particular the influence of the lipid bilayer on TCR-pMHC interactions. Since the MHC and TCR are membrane-embedded proteins, the influence of the membrane environment itself is an essential ingredient for TCR-pMHC interaction and T cell response [44]. Various advanced MD studies have been performed to evaluate the role of the lipid bilayer for TCR-pMHCII systems: In a ground-breaking study, Wan et al. [45] performed MD simulations of a membrane-embedded TCR-pMHC-CD4 complex. Although the duration of the simulation was limited to 10 ns due to the size of the system (more than 300.000 atoms), the computed structural and thermodynamic properties, such as binding free energies of TCR-pMHC and pMHC-CD4, were in fair agreement with experimental data. Bello et al. [46] studied the influence of the membrane on the dynamics and energetics of peptide-bound and peptide-free MHC class II molecules in aqueous and membrane-bound environments by 100 and 150 ns MD simulations and found that the presence of the membrane might restrict the conformational flexibility of the peptide-binding cleft. More recently, Bello et al. [47] explored the energetic and dynamic behavior of a pMHCII-TCR complex embedded in two opposing lipid membranes using three independent 300 ns-long unbiased MD simulations. This study provided evidence of the main contributors to the pMHCII-TCR complex formation and identified key residues involved in this molecular recognition process.

The formation of the immunological synapse incorporates co-receptors, integrins, and various signaling proteins that trigger signaling cascades resulting in T-cell activation. Therefore, future MD studies of the 3KPS system should not only include the lipid membrane, but also co-receptors (CD8, CD3 complex) and other co-stimulatory molecules to assess their influence on the system [6].

Another limitation of the present study relates to the simulation time of 40 ns, which is way too short to reliably cover all processes involved, e.g., in TCR-signaling or even dissociation of the TCR-pMHC complex. Several authors have used coarse-grained simulations and elastic network models for modeling protein flexibility and interactions, see the recent reviews by Kmiecik et al. [48,49]. Moreover, steered MD [50] has been applied to extend simulation times to more realistic regimes.

## 5. Conclusions

We have performed MD simulations with different starting configurations to monitor differences in intramolecular domain movements between bound and unbound TCR-pMHC molecules of the LC13-HLA-B*44:05-pEEYLQAFTY complex. The results provide some insights into alterations of the dynamics as a consequence of complex formation: For the domains MHC α1 and MHC α2 the angles between corresponding eigenvectors show larger variation in the unbound state, indicating a wider range of the groove between the two α-helices. For the TCR V_α_ and TCR V_β_ domains, angles between corresponding eigenvectors indicate little movements in their main orientation but significant rotations around these major axes. The MHC α2-helix showed larger RMSD flexibility for unbound states. The observed structural changes in the CDR3α loop upon binding together with the large RMSF-values of the same region in the unbound state (see Figure 12A) are consistent with the view that structural changes involved in protein binding correlate with intrinsic motions of proteins in the unbound state [51]. Moreover, the structural transition from a 3_10_-helix to a coil in the CDR3α loop upon complex formation supports the hypothesis that (at least for the CDR3α loop) TCR-pMHC binding can be characterized by the induced fit model, whereby structural rearrangements of the TCR CDRs ensure to achieve the most stable complex [3]. Differences in conformational flexibility (as characterized by RMSD- and RMSF-values) between free and bound forms both for the MHC α-helices and the peptide are consistent with the view that the TCR perceives the peptide and the MHC as a single, composite ligand, making only little distinction between them [7]. Although even our 30 relatively short MD simulations of 40 ns each revealed interesting dynamical features not available from crystallographic data alone, the length of simulations should be extended in subsequent studies to cover processes with longer characteristic time scales.

## Figures and Tables

**Table 1:** RMSD (mean ± SD) in nm for various domains of the unbound molecules TCR and pMHC as calculated from 20 (10 for free TCR and 10 for free MHC) 40 ns MD runs (1–10) and from two (free TCR and free MHC) 400 ns MD runs (11).

Run	TCR V_α_	TCR V_β_	MHC α1	MHC α2	MHC Peptide
1	0.101 ± 0.013	0.109 ± 0.017	0.087 ± 0.019	0.132 ± 0.021	0.157 ± 0.015
2	0.106 ± 0.011	0.084 ± 0.009	0.059 ± 0.013	0.111 ± 0.014	0.061 ± 0.017
3	0.112 ± 0.015	0.083 ± 0.011	0.075 ± 0.011	0.079 ± 0.011	0.123 ± 0.012
4	0.126 ± 0.010	0.087 ± 0.011	0.061 ± 0.010	0.117 ± 0.019	0.117 ± 0.034
5	0.114 ± 0.016	0.087 ± 0.009	0.066 ± 0.009	0.079 ± 0.019	0.085 ± 0.017
6	0.102 ± 0.011	0.093 ± 0.014	0.083 ± 0.011	0.153 ± 0.039	0.096 ± 0.020
7	0.129 ± 0.011	0.119 ± 0.011	0.061 ± 0.010	0.097 ± 0.013	0.085 ± 0.014
8	0.114 ± 0.011	0.087 ± 0.010	0.084 ± 0.016	0.073 ± 0.013	0.104 ± 0.020
9	0.118 ± 0.011	0.122 ± 0.016	0.076 ± 0.012	0.118 ± 0.031	0.064 ± 0.018
10	0.124 ± 0.009	0.091 ± 0.010	0.080 ± 0.015	0.107 ± 0.016	0.094 ± 0.014
11	0.108 ± 0.016	0.085 ± 0.013	0.077 ± 0.013	0.067 ± 0.015	0.085 ± 0.011

**Table 2:** RMSD (mean ± SD) in nm for various domains of the bound complex TCR-pMHC as calculated from ten 40 ns MD runs (1–10) and from one 144 ns MD run (11).

Run	TCR V_α_	TCR V_β_	MHC α1	MHC α2	MHC Peptide
1	0.099 ± 0.010	0.071 ± 0.007	0.074 ± 0.009	0.076 ± 0.010	0.052 ± 0.010
2	0.098 ± 0.008	0.069 ± 0.008	0.070 ± 0.009	0.094 ± 0.011	0.056 ± 0.007
3	0.101 ± 0.011	0.087 ± 0.018	0.093 ± 0.014	0.095 ± 0.022	0.048 ± 0.007
4	0.111 ± 0.010	0.069 ± 0.007	0.070 ± 0.015	0.127 ± 0.024	0.082 ± 0.015
5	0.098 ± 0.009	0.080 ± 0.012	0.076 ± 0.007	0.072 ± 0.009	0.046 ± 0.006
6	0.090 ± 0.008	0.064 ± 0.007	0.079 ± 0.011	0.120 ± 0.022	0.047 ± 0.006
7	0.100 ± 0.013	0.068 ± 0.007	0.084 ± 0.013	0.117 ± 0.028	0.052 ± 0.011
8	0.105 ± 0.009	0.067 ± 0.008	0.084 ± 0.014	0.134 ± 0.021	0.057 ± 0.011
9	0.100 ± 0.011	0.070 ± 0.008	0.076 ± 0.011	0.079 ± 0.014	0.057 ± 0.016
10	0.109 ± 0.016	0.066 ± 0.006	0.067 ± 0.016	0.105 ± 0.029	0.083 ± 0.011
11	0.097 ± 0.011	0.073 ± 0.010	0.073 ± 0.013	0.081 ± 0.012	0.066 ± 0.008

## Data Availability

For this study data have been downloaded from the publically available protein data bank (https://www.rcsb.org/).

## Supplementary Materials

The following are available online at https://www.mdpi.com/2073-4409/8/7/720/s1, Figure S1: Cartoon representations of CDR3α loops (Ile91, Leu92, Pro93, Leu94, Ala95, Gly96, Gly97, Thr98, Ser99, Tyr100, Gly102, Lys103, Leu104, Thr105) in free (A) and bound (B) states. Residues Ala95 (blue), Gly96 (grey), and Gly97 (grey) exhibit a structural change from a 3_10_-helix (free, panel A) to a coil (bound, panel B). VMD [22] was used to identify structural changes and to produce the figures. Video S1: Movie of one 144 ns run of the TCR-pMHC complex started from the crystal structure 3KPS.

## References

[1] Rudolph M.G., Wilson I.A. (2002). The specificity of TCR/pMHC interaction. Curr. Opin. Immunol..

[2] Garcia K.C., Degano M., Stanfield R.L., Brunmark A., Jackson M.R., Peterson P.A., Teyton L., Wilson I.A. (1996). An alphabeta T cell receptor structure at 2.5 A and its orientation in the TCR-MHC complex. Science.

[3] Armstrong K.M., Piepenbrink K.H., Baker B.M. (2008). Conformational changes and flexibility in T-cell receptor recognition of peptide-MHC complexes. Biochem. J..

[4] Rudolph M.G., Stanfield R.L., Wilson I.A. (2006). How TCRs bind MHCs, peptides, and coreceptors. Annu. Rev. Immunol..

[5] Bjorkman P.J. (1997). MHC restriction in three dimensions: A view of T cell receptor/ligand interactions. Cell.

[6] Kass I., Buckle A.M., Borg N.A. (2014). Understanding the structural dynamics of TCR-pMHC complex interactions. Trends Immunol..

[7] Baker B.M., Scott D.R., Blevins S.J., Hawse W.F. (2012). Structural and dynamic control of T-cell receptor specificity, cross-reactivity, and binding mechanism. Immunol. Rev..

[8] Reiser J.B., Gregoire C., Darnault C., Mosser T., Guimezanes A., Schmitt-Verhulst A.M., Fontecilla-Camps J.C., Mazza G., Malissen B., Housset D. (2002). A T cell receptor CDR3beta loop undergoes conformational changes of unprecedented magnitude upon binding to a peptide/MHC class I complex. Immunity.

[9] Ma Z., Janmey P.A., Finkel T.H. (2008). The receptor deformation model of TCR triggering. FASEB J..

[10] Choudhuri K., Van Der Merwe P.A. (2007). Molecular mechanisms involved in T cell receptor triggering. Semin. Immunol..

[11] Ribarics R., Kenn M., Karch R., Ilieva N., Schreiner W. (2015). Geometry Dynamics of Alpha- Helices in Different Class I Major Histocompatibility Complexes. J. Immunol. Res..

[12] Zacharias M., Springer S. (2004). Conformational flexibility of the MHC class I alpha1-alpha2 domain in peptide bound and free states: A molecular dynamics simulation study. Biophys. J..

[13] Toh H., Kamikawaji N., Tana T., Sasazuki T., Kuhara S. (1998). Molecular dynamics simulations of HLA-DR4 (DRB1*0405) complexed with analogue peptide: Conformational changes in the putative T-cell receptor binding regions. Protein Eng..

[14] Reboul C.F., Meyer G.R., Porebski B.T., Borg N.A., Buckle A.M. (2012). Epitope flexibility and dynamic footprint revealed by molecular dynamics of a pMHC-TCR complex. PLoS Comput. Biol..

[15] Wolfson M.Y., Nam K., Chakraborty A.K. (2011). The effect of mutations on the alloreactive T cell receptor/peptide-MHC interface structure: A molecular dynamics study. J. Phys. Chem. B.

[16] Stavrakoudis A. (2011). Insights into the structure of the LC13 TCR/HLA-B8-EBV peptide complex with molecular dynamics simulations. Cell Biochem. Biophys..

[17] Tedeschi V., Alba J., Paladini F., Paroli M., Cauli A., Mathieu A., Sorrentino R., D’Abramo M., Fiorillo T.M. (2019). Unusual Placement of an EBV Epitope into the Groove of the Ankylosing Spondylitis-Associated HLA-B27 Allele Allows CD8+ T Cell Activation. Cells.

[18] Macdonald W.A., Chen Z., Gras S., Archbold J.K., Tynan F.E., Clements C.S., Bharadwaj M., Kjer-Nielsen L., Saunders P.M., Wilce M.C. (2009). T Cell Allorecognition via Molecular Mimicry. Immunity.

[19] Ferber M., Zoete V., Michielin O. (2012). T-cell receptors binding orientation over peptide/MHC class I is driven by long-range interactions. PLoS ONE.

[20] Ribarics R., Karch R., Ilieva N., Schreiner W. (2014). Geometric analysis of alloreactive HLA α-helices. Biomed Res. Int..

[21] Kenn M., Ribarics R., Ilieva N., Schreiner W. (2014). Finding semirigid domains in biomolecules by clustering pair-distance variations. Biomed Res. Int..

[22] Humphrey W., Dalke A., Schulten K. (1996). VMD: Visual molecular dynamics. J. Mol. Graph..

[23] Hess B., Kutzner C., van der Spoel D., Lindahl E. (2008). GROMACS 4: Algorithms for Highly Efficient, Load-Balanced, and Scalable Molecular Simulation. J. Chem. Theory Comput..

[24] Lindorff-Larsen K., Piana S., Palmo K., Maragakis P., Klepeis J.L., Dror R.O., Shaw D.E. (2010). Improved side-chain torsion potentials for the Amber ff99SB protein force field. Proteins.

[25] Berendsen H.J.C., Postma J.P.M., Van Gunsteren W.F., Hermans J. (1981). Interaction models for water in relation to protein hydration. Intermolecular Forces.

[26] Amadei A., Linssen A.B.M., Berendsen H.J. (1993). Essential Dynamics of Proteins. Proteins.

[27] Kim H.J., Choi M.Y., Kim H.J., Llinás M. (2010). Conformational Dynamics and Ligand Binding in the Multi-Domain Protein PDC109. PLoS ONE.

[28] Hub J.S., de Groot B.L. (2009). Detection of functional modes in protein dynamics. PLoS Comput. Biol..

[29] Schreiner W., Karch R., Ribarics R., Cibena M., Ilieva N. (2015). Relative Movements of Domains in Large Molecules of the Immune System. J. Immunol. Res..

[30] Gakamsky D.M., Luescher I.F., Pecht I. (2004). T cell receptor-ligand interactions: A conformational preequilibrium or an induced fit. Proc. Natl. Acad. Sci. USA.

[31] Ahmad M., Gu W., Geyer T., Helms V. (2011). Adhesive water networks facilitate binding of protein interfaces. Nat. Commun..

[32] Lee J.K., Stewart-Jones G., Dong T., Harlos K., Di Gleria K., Dorrell L., Douek D.C., van der Merwe P.A., Jones E.Y., McMichael A.J. (2004). T cell cross-reactivity and conformational changes during TCR engagement. J. Exp. Med..

[33] Borbulevych O.Y., Piepenbrink K.H., Gloor B.E., Scott D.R., Sommese R.F., Cole D.K., Sewell A.K., Baker B.M. (2009). T cell receptor cross-reactivity directed by antigen-dependent tuning of peptide-MHC molecular flexibility. Immunity.

[34] Borbulevych O.Y., Piepenbrink K.H., Baker B.M. (2011). Conformational melding permits a conserved binding geometry in TCR recognition of foreign and self molecular mimics. J. Immunol..

[35] Madden D.R., Garboczi D.N., Wiley D.C. (1993). The antigenic identity of peptide-MHC complexes: A comparison of the conformations of five viral peptides presented by HLA-A2. Cell.

[36] Tynan F.E., Burrows S.R., Buckle A.M., Clements C.S., Borg N.A., Miles J.J., Beddoe T., Whisstock J.C., Wilce M.C., Silins S.L. (2005). T cell receptor recognition of a ‘super-bulged’ major histocompatibility complex class I-bound peptide. Nat. Immunol..

[37] Wieczorek M., Abualrous E.T., Sticht J., Alvaro-Benito M., Stolzenberg S., Noé F., Freund C. (2017). Major Histocompatibility Complex (MHC) Class I and MHC Class II Proteins: Conformational Plasticity in Antigen Presentation. Front. Immunol..

[38] Ayres C.M., Corcelli S.A., Baker B.M. (2017). Peptide and Peptide-Dependent Motions in MHC Proteins: Immunological Implications and Biophysical Underpinnings. Front. Immunol..

[39] Natarajan K., Jiang J., May N.A., Mage M.G., Boyd L.F., McShan A.C., Sgourakis N.G., Bax A., Margulies D.H. (2018). The Role of Molecular Flexibility in Antigen Presentation and T Cell Receptor-Mediated Signaling. Front. Immunol..

[40] Hawse W.F., Champion M.M., Joyce M.V., Hellman L.M., Hossain M., Ryan V., Pierce B.G., Weng Z., Baker B.M. (2012). Cutting edge: Evidence for a dynamically driven T cell signaling mechanism. J. Immunol..

[41] Rossjohn J., Gras S., Miles J.J., Turner S.J., Godfrey D.I., McCluskey J. (2015). T cell antigen receptor recognition of antigen-presenting molecules. Annu. Rev. Immunol..

[42] Hoffmann T., Krackhardt A.M., Antes I. (2015). Quantitative Analysis of the Association Angle between T-cell Receptor Vα/Vβ Domains Reveals Important Features for Epitope Recognition. PLoS Comput. Biol..

[43] Adler M., Beroza P. (2013). Improved Ligand Binding Energies Derived from Molecular Dynamics: Replicate Sampling Enhances the Search of Conformational Space. J. Chem. Inf. Model..

[44] He H.T., Bongrand P. (2012). Membrane dynamics shape TCR-generated signaling. Front. Immunol..

[45] Wan S., Flower D.R., Coveney P.V. (2008). Toward an atomistic understanding of the immune synapse: Large-scale molecular dynamics simulation of a membrane-embedded TCR-pMHC-CD4 complex. Mol. Immunol..

[46] Bello M., Correa-Basurto J. (2013). Molecular dynamics simulations to provide insights into epitopes coupled to the soluble and membrane-bound MHC-II complexes. PLoS ONE.

[47] Bello M., Correa-Basurto J. (2016). Energetic and flexibility properties captured by long molecular dynamics simulations of a membrane-embedded pMHCII-TCR complex. Mol. Biosyst..

[48] Kmiecik S., Kouza M.A.O., Badaczewska-Dawid A.E., Kloczkowski A., Kolinski A.A.O. (2018). Modeling of Protein Structural Flexibility and Large-Scale Dynamics: Coarse-Grained Simulations and Elastic Network Models. Int. J. Mol. Sci..

[49] Kmiecik S., Gront D., Kolinski M., Wieteska L., Dawid A.E., Kolinski A. (2016). Coarse-Grained Protein Models and Their Applications. Chem. Rev..

[50] Cuendet M.A., Michielin O. (2008). Protein-protein interaction investigated by steered molecular dynamics: The TCR-pMHC complex. Biophys. J..

[51] Tobi D., Bahar I. (2005). Structural changes involved in protein binding correlate with intrinsic motions of proteins in the unbound state. Proc. Natl. Acad. Sci. USA.

