# Supplementary material for: Intramolecular Domain Movements of Free and Bound pMHC and TCR Proteins: A Molecular Dynamics Simulation Study
Source: Cells. 2019 Jul 13;8(7):720. doi: 10.3390/cells8070720 (PMC6678086; doi:10.3390/cells8070720)
Supplement: Supplementary file 1 [file cells-08-00720-s001.zip › cells-528544-revision3-SupplementaryFigureS1.pdf]

Supplementary material

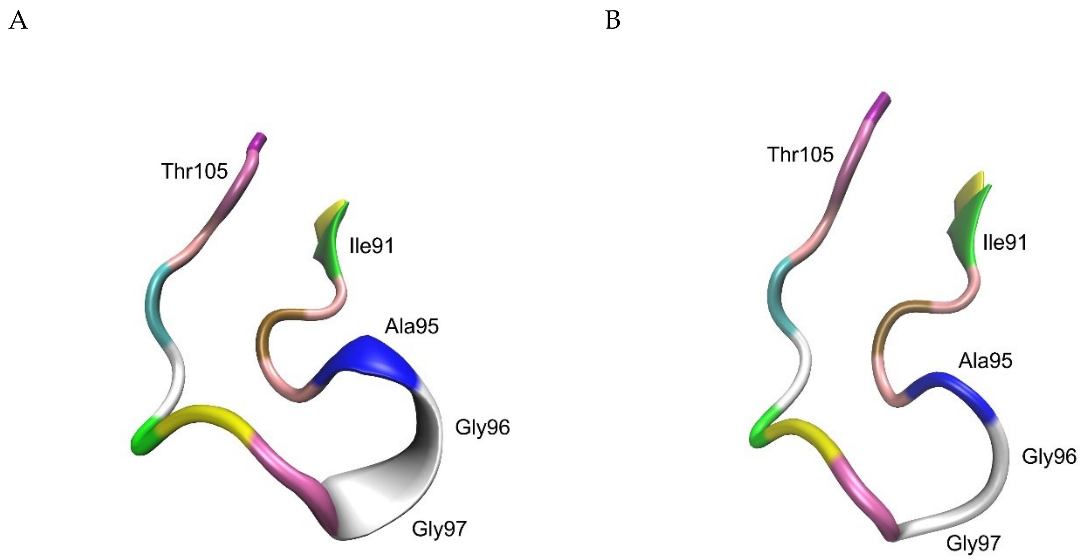

Figure S1. Cartoon representations of CDR3α loops (Ile91, Leu92, Pro93, Leu94, Ala95, Gly96, Gly97, Thr98, Ser99, Tyr100, Gly102, Lys103, Leu104, Thr105) in free (**A**) and bound (**B**) states. Residues Ala95 (blue), Gly96 (grey), and Gly97 (grey) exhibit a structural change from a 3_{10}-helix (free, panel **A**) to a coil (bound, panel **B**). VMD [1] was used to identify structural changes and to produce the figures.

References

1. Humphrey, W.; Dalke, A.; Schulten, K. VMD: visual molecular dynamics. *J. Mol. Graph.* **1996**, *14*, 33-38, 27-38.
